# Screening for neurodevelopmental disorders in preterm Infants

**DOI:** 10.1192/j.eurpsy.2025.1237

**Published:** 2025-08-26

**Authors:** N. Zaidi, D. Ben Touhemie, K. Khemekhem, K. Chiha, J. Boudabous, I. Hadjkacem, A. Gargouri, H. Ayédi, N. Ben Hmida, Y. Moalla

**Affiliations:** 1 pédosychiatrie; 2 néonatologie, Hédi Chaker university hospital, Sfax, Tunisia

## Abstract

**Introduction:**

Early detection of neurodevelopmental disorders (NDD) allows for timely intervention and tailored support to address behavioral and learning issues and reduce the impact of severe disabilities. Certain populations of newborns, particularly premature infants, are at higher risk for developing such disorders; identifying them enables the establishment of preventive monitoring and screening.

**Objectives:**

to evaluate the frequency of neurodevelopmental disorders (NDD) in preterm children seen at the outpatient Neonatology department

**Methods:**

Our study was a descriptive cross-sectional study conducted on 193 premature infants hospitalized in the Neonatology service. All children underwent at least two psychiatric assessments evaluating child development, early interactions, and behavioral problems. These assessments were conducted at the Neonatology outpatient department by a child psychiatrist in the presence of at least one parent, from 2016 to June 2024. Sociodemographic and clinical data were collected from their medical records.

**Results:**

In our sample, the average age of infants was 2 years, with a range from 10 months to 3 years. The sex ratio was 1:1. According to the psychiatric interview and clinical examination, we found that 74.1% of children exhibited psychomotor developmental delays, 15% of them have global developmental delays, 11.4% of children have language delays, and 6.7% of them have been diagnosed with autism spectrum disorder, according to DSM-5 criteria. Among the assessed children, 6.7% showed behavioral issues, and 2.5% presented with reactive attachment disorders. Among the examined children, 24.4% were referred for regular follow-up at the outpatient child psychiatry clinic.

**Conclusions:**

Our study indicates that preterm infants are at a high risk of developing neurodevelopmental disorders due to the immaturity and vulnerability of their brains during critical developmental periods. These findings underscore the importance of Child Psychiatry examination for this population.

**Disclosure of Interest:**

None Declared

